# Sulfated Cholecystokinin-8 Promotes CD36—Mediated Fatty Acid Uptake into Primary Mouse Duodenal Enterocytes

**DOI:** 10.3389/fphys.2017.00660

**Published:** 2017-09-01

**Authors:** Claire Demenis, John McLaughlin, Craig P. Smith

**Affiliations:** School of Medical Sciences, University of Manchester Manchester, United Kingdom

**Keywords:** enteroendocrine cells, duodenum, cholecystokinin, GLP-1, GLP-2, oleoylethanolamine

## Abstract

Cholecystokinin (CCK) is an archetypal incretin hormone secreted by intestinal enteroendocrine cells (EEC) in response to ingested nutrients. The aim of this study was to determine whether CCK modulates enterocyte fatty acid uptake by primary mouse duodenal cells. Exposure of primary mouse duodenal cells to 10 pM sulfated CCK-8 caused a two fold increase in dodecanoic acid fatty acid (FA) uptake. The selective CCK A receptor antagonist loxiglumide (100 μM) completely abolished the CCK-8 induced FA uptake. The CD36 fatty acid translocase-specific inhibitor sulfo-N-succinimidyl oleate (1 μM) also completely inhibited CCK-8 induced FA uptake, as did treatment with 200 μM phloretin. Together these data show CCK induces FA uptake into duodenal enterocytes; this action involves the CCK-R_A_ receptor and is carrier mediated by CD36.

## Introduction

In the proximal intestine peptide hormones are released by enteroendocrine cells (EEC) in response to ingested nutrients, and function to orchestrate digestion in order to maximize nutrient absorption and utilization. Several so-called gut hormones have independently been shown to act directly on enterocytes to modulate nutrient absorption. In particular, the gastrointestinal peptide, GLP-2, and the ethanolamide lipid, Oleoylethanolamine (OEA) have each been reported to modulate fatty acid (FA) uptake by enterocytes (Yang et al., 2007; Hsieh et al., 2009). Conversely GLP-1, a product of the same gene as GLP-2, has been shown to decrease intestinal FA absorption (Hsieh et al., 2009; Mellitzer and Gradwohl, 2011).

The mechanism responsible for GLP-2—stimulated absorption involves increased expression and activity of the FA-sensing receptors cluster of differentiation CD36 protein (CD36); a membrane bound protein that potentiates cellular fatty acid uptake (Hsieh et al., 2009; Mellitzer and Gradwohl, 2011). Interestingly, CD36 has also been reported to be involved in orosensory, intestinal, and neuronal sensing of FA (Laugerette et al., 2005; Schwartz et al., 2008).

Cholecystokinin (CCK), often referred to as the archetypal gut hormone, is a key intestinal peptide secreted by small intestinal enteroendocrine cells in response to ingested nutrients (McLaughlin et al., 1998, 1999; Dockray, 2012; Rehfeld, 2017). Release of CCK into the circulation is primarily triggered by the presence of fatty acids or peptides in the intestinal lumen (Liddle et al., 1986; Cordier-Bussat et al., 1997; McLaughlin et al., 1999). Membrane-bound G protein coupled receptors resident on enteroendocrine cells transduce nutrient signals resulting in activation of second messenger cascades and secretion of CCK (Liou et al., 2011).

CCK is synthesized as a 115 amino acid prepro hormone, processed, then released in several forms that vary in peptide chain length and which are derived from a 58 residue peptide. The reported bioactivity of CCK resides in the C-terminal heptapeptide (Rehfeld, 2017). CCK's several actions are geared to promoting digestion of nutrients in particular ingested fatty acids. Once released, CCK slows gastric emptying, stimulates secretion of pancreatic digestive enzymes, and stimulates the release of bile by triggering contraction of the gall bladder and relaxation of the sphincter of Oddi. Centrally, CCK induces satiety, an action mediated via stimulation of the vagus nerve (Dockray, 2012). These actions are mediated by CCK receptors, of which two have been characterized: CCK-R_A_ and CCK-R_B_ (Kopin et al., 1992; Noble et al., 1999). CCK-R_A_ has approximately 100-fold higher affinity for CCK over gastrin compared to the CCK-R_B_ (Kopin et al., 1992; Wank et al., 1992; Dufresne et al., 2006).

Surprisingly, there is a scarcity of published research pertaining to a possible action of CCK to promote nutrient uptake, although indirect evidence is apparent that may point to this action (e.g., King et al., 2015). Given that CCK is secreted in response to fats we reasoned that CCK, like GLP-2 or OEA, may induce effects that facilitate FA absorption (McLaughlin et al., 1999). Therefore, the aim of the research described herein was to determine whether CCK modulates FA uptake into mouse primary duodenal enterocytes and if so to determine the mechanism responsible for this action. To assess these proposals we developed a robust fluorimetric assay and measured uptake of labeled-FA into mouse primary enterocytes.

## Materials and methods

### Experimental animals

Unless otherwise stated all experiments were performed on cells isolated from male C57BL/6N (strain code 027) mice purchased from Envigo, UK. Where stated, male CCK-eGFP (Tg(CCKEGFP)BJ203Gsat/Mmmh; Gong et al., 2003) and male CCK ^ko/LacZ^ transgenic mice (Lacourse et al., 1999) were also used. Initially CCK-eGFP founder mice were purchased from MMRRC (Mutant Mouse Regional Resource Center, USA) and bred in-house at Manchester. CCK ^ko/LacZ^ have the CCK locus replaced by a knock-in LacZ cassette, rendering homozygotes of the transgene devoid of CCK (Lacourse et al., 1999). All mice were subject to a 12:12-h light/dark cycle and fed *ad libitum* with standard chow diet. Adult mice (8–12 weeks old) were used for all experiments. Mice were killed by CO_2_ asphyxiation and duodenums removed. The University of Manchester Ethical Review Process Committee, in accordance with the UK Home Office regulations, under license 40/3409, ethically approved all animal procedures. All animal procedures undertaken in this study were in accordance with Animals Scientific Procedures Act 1986 (UK) and UK Home Office regulations.

### Preparation of dissociation duodenal cells

The cell disassociation protocol was as described by Sykaras et al. and has been demonstrated to successfully isolate intestinal epithelial cells whilst maintaining cell viability (Sykaras et al., 2012). Dissociated cells were maintained at 37°C in HBSS (pH 7.4) supplemented with 1.2 mM CaCl_2_, 10 mM HEPES, 5 mM glucose, 10% FBS and gassed with 5% CO_2_ for no more than 1 h prior to experimentation.

C12 Bodipy fluorescent fatty acid (Bodipy-FA, Invitrogen, UK) was utilized to determine the dynamics of fatty acid uptake in intestinal enterocytes. The Bodipy-FA uptake protocol was based on methods previously described by others (Gimeno et al., 2003; Yang et al., 2007; Lynes et al., 2011). Preliminary experiments were performed to derive the optimal incubation time and Bodipy-FA concentration (see Supplementary Figure 1) and an incubation time of 2 min and a Bodipy-FA concentration of 5 μM were chosen.

The optimized FA uptake protocol was as follows: Aliquots of dissociated duodenal cells were centrifuged at 800 g RCF for 5 min, then cells were washed by gentle trituration in Dulbecco's complete PBS containing 0.9 mM Ca^2+^ and 0.5 mM Mg^2+^, referred hereafter as DPBS. Cells were repelleted and then used for assessment of FA uptake. To measure FA uptake cells were resuspended in 500 μl DPBS containing 10 μM fatty acid free BSA (Sigma Aldrich, UK) and 5 μM 4,4-difluoro-5-methyl-4-bora-3a,4a-diaza-s-indacene-3-dodecanoic acid (BODIPY(R) 500/510 C1, C12) Bodipy fluorescent fatty acids giving a BSA: Bodipy-FA ratio of 2.1. Prior to its addition to cells, the Bodipy-FA solution was sonicated for 15 min to evenly disperse fatty acids. Uptake experiments were carried out in the dark, to protect fluorescence integrity of Bodipy and cells were incubated in Bodipy-FA solution for 2 min and then centrifuged (800 g RCF) at 4°C for 5 min. The resultant supernatant was discarded and cell pellets were gently dispersed in 500 μl ice-cold “stop buffer” consisting of DPBS, 1% fatty acid free BSA and 200 μM phloretin (Sigma Aldrich, UK) and centrifuged (2 min, 800 g RCF). Following a further cycle of resuspension and centrifugation, cell pellets were resuspended in 500 μl of stop buffer and kept in the dark on ice until cellular Bodipy fluorescence was measured by flow cytometry. All experimental replicates were performed in triplicate.

### Flow cytometric analysis of cellular uptake of bodipy fatty acids

Cellular fluorescence was measured using a Beckman Coulter CyAn ADP cell sorter running Summit software (version 4.3). A 488 nm laser was used for excitation and fluorescent signal was detected as a 530/30 nm band pass. Fluorescence was measured for 10,000 events from each sample replicate and a mean value was obtained for three replicates per treatment group. To enable gating parameters to be set, control cells were also analyzed in the absence of Bodipy-FA (control samples, see Supplementary Figures 2, 3).

### Measuring the effect of test substances on Bodipy-FA uptake into mouse duodenal cells

To test the effect of CCK concentration on cellular Bodipy-FA uptake, cells were incubated with CCK-8 (sulfated or unsulfated, Tocris, UK) over a concentration range of 10^−12^–10^−8^ M for 15 min prior to exposure to Bodipy-FA. In parallel and as a positive control for hormone-stimulated FA uptake, cells were treated with either 10 pM GLP-2 or 10 nM OEA. To test the involvement of the CCK-R_A_ in the CCK-induced response the CCK-R_A_-specific inhibitor loxiglumide (Sigma Aldrich, UK) was employed. Cells were prepared as described and Bodipy-FA uptake measured in the presence of 100 μM loxiglumide. Additional controls were performed using 50-fold excess (250 μM) of unlabeled decanoic acid (Sigma Aldrich, UK) to confirm the fidelity of the Bodipy-FA as a surrogate marker for fatty acid uptake studies (Supplementary Figure 4).

To determine if Bodipy-FA uptake was carrier mediated, two well-characterized inhibitors of membrane FA transporters were employed: Phloretin (200 μM), a broad spectrum inhibitor of protein mediated FA transport, including that mediated by CD36 (Abumrad et al., 1981; Ibrahimi et al., 1996), or the CD36-specific inhibitor sulfo-N-succinimidyl oleate (1 μM SSO; Coort et al., 2002; Pohl et al., 2005; Schwenk et al., 2010). Cells were incubated for 15 min with either phloretin (200 μM) or SSO (1 μM) prior to addition of Bodipy-FA and exposure to CCK-8. SSO was a kind gift from Prof. Glatz, Maastricht University, The Netherlands.

### Data processing and statistical analysis

Measurements were made in triplicate on each pool of cells isolated from a single mouse and for each sample a mean fluorescence was calculated for 10,000 events. Statistics were then performed using the mean fluorescence of each replicate of which there were three replicates per treatment and means and SEM calculated. Measurements were normalized to the control (untreated cells exposed to Bodipy-FA. Pools of cells derived from a single mouse acted as control or were exposed to hormone. Complete experiments were repeated three times using different mice. Statistical analysis was carried out using GraphPad Prism for Mac, Version 6. Sample mean values are compared to mean control values using an Analysis of Variance (ANOVA) followed by a *post-hoc* Dunnets test. When comparing pre-selected pairs of data, the ANOVA test was performed, followed by the *post-hoc* Bonferroni test. When comparing only two sets of mean values an unpaired *t*-test was used. In all cases *P*-values ≤0.05 were considered significant.

## Results

### CCK increases enterocyte fatty acid uptake

**Figure 1:**
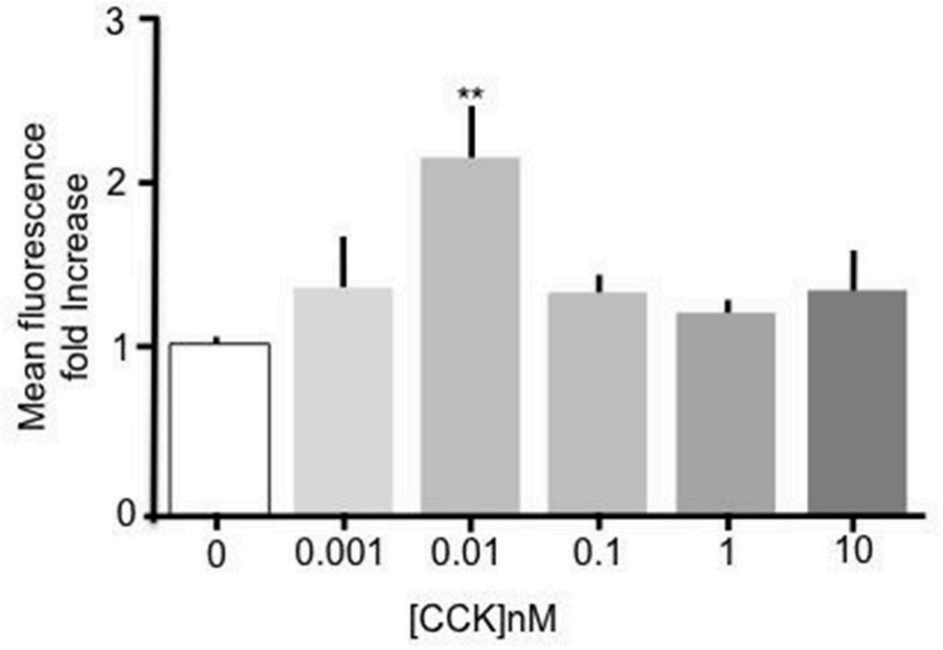
CCK stimulated uptake of Bodipy-FA by primary duodenal cells. Sulfated CCK-8 concentration response experiment. Intestinal cells were incubated for 15 min in concentrations of CCK ranging from 10^−8^ to 10^−12^ M. Cells were then incubated with Bodipy-FA for 2 min. 10 pM CCK caused a Significant (*p* > 0.01) increase in cell fluorescence. Means represent replicates performed in triplicate and error bars represent SEM (*n* = 3) ^**^p > 0.01 compared to control.

Incubation of primary mouse enterocytes with sulfated CCK-8 over a range of concentrations (1 pM–10 nM) showed that CCK induced a significant increase in Bodipy-FA uptake (Figure 1). Compared to unstimulated control cells, treatment with 10 pM CCK caused a doubling of Bodipy-FA uptake (*p* < 0.01). Although there was an indication of dose dependent activation only treatment with 10 pM CCK caused a significant increase in Bodipy-FA uptake. In addition, Bodipy-FA uptake remained unchanged compared to unstimulated control cells when the concentration-effect experiment was repeated using unsulfated CCK-8 instead of sulfated CCK (data not shown). In summary, like GLP-2 and OEA, sulfated CCK-8 induces increased uptake of fatty acids in duodenal epithelial cells.

**Figure 2:**
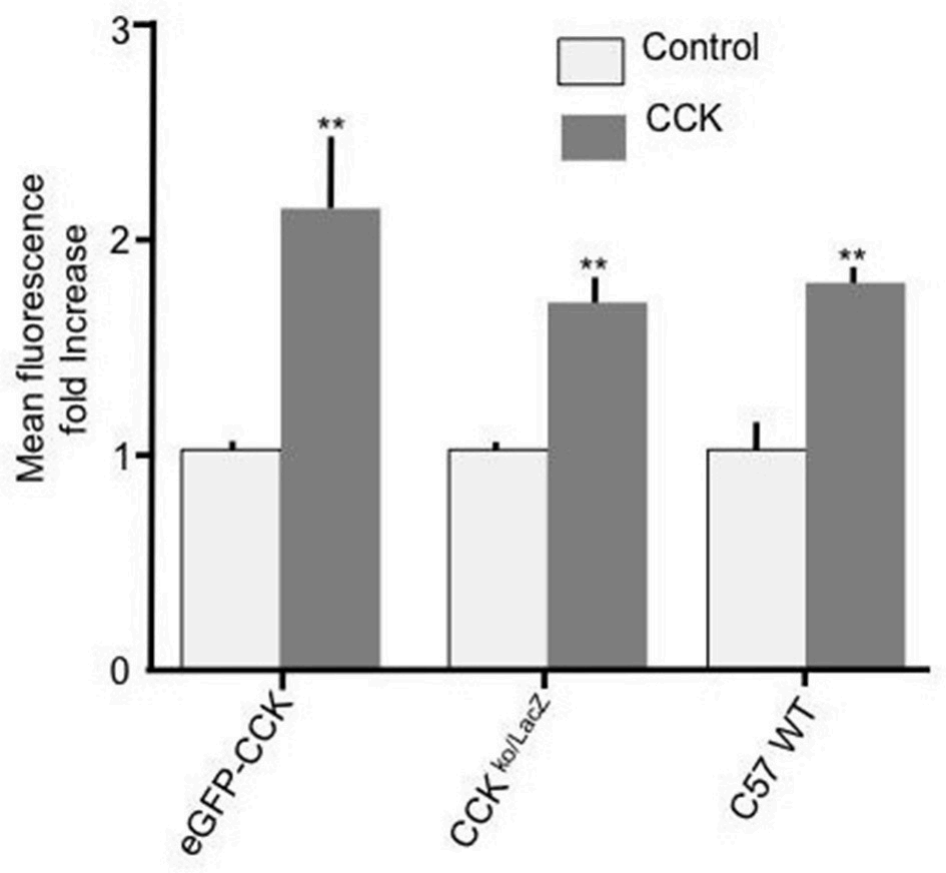
CCK stimulates cellular FA uptake irrespective of mouse strain. Incubation of duodenal cells with 10 pM CCK caused a significant increase in Bodipy-FA uptake into cells from eGFP-CCK mice, CCK ^ko/LacZ^ mice or C57 WT mice. Each data set was normalized to respective control mean to enable direct comparisons. Means represent replicates performed in triplicate and error bars represent SEM (*n* = 3). ^**^p > 0.01 compared with corresponding control.

To further confirm that CCK has a stimulatory effect on enterocyte fatty acid uptake and to assure that the observed effect was not a consequence of a particular genetic background, duodenal enterocytes from two independent mouse strains with different genetic backgrounds to C57BL/6N (strain code 027), namely, CCK-eGFP (mixed background: 129S2/SvPas ^*^ C57BL/6J ^*^ FVB/NTac ^*^ Swiss Webster) and CCK ko/LacZ (129X1/SvJ background) were prepared and tested. In both mouse strains, 10 pM CCK caused a significant increase in Bodipy-FA uptake (*p* < 0.01, Figure 2). Therefore, the stimulatory effect of CCK on fatty acid uptake was evident in mouse strains of dissimilar genetic backgrounds.

**Figure 3:**
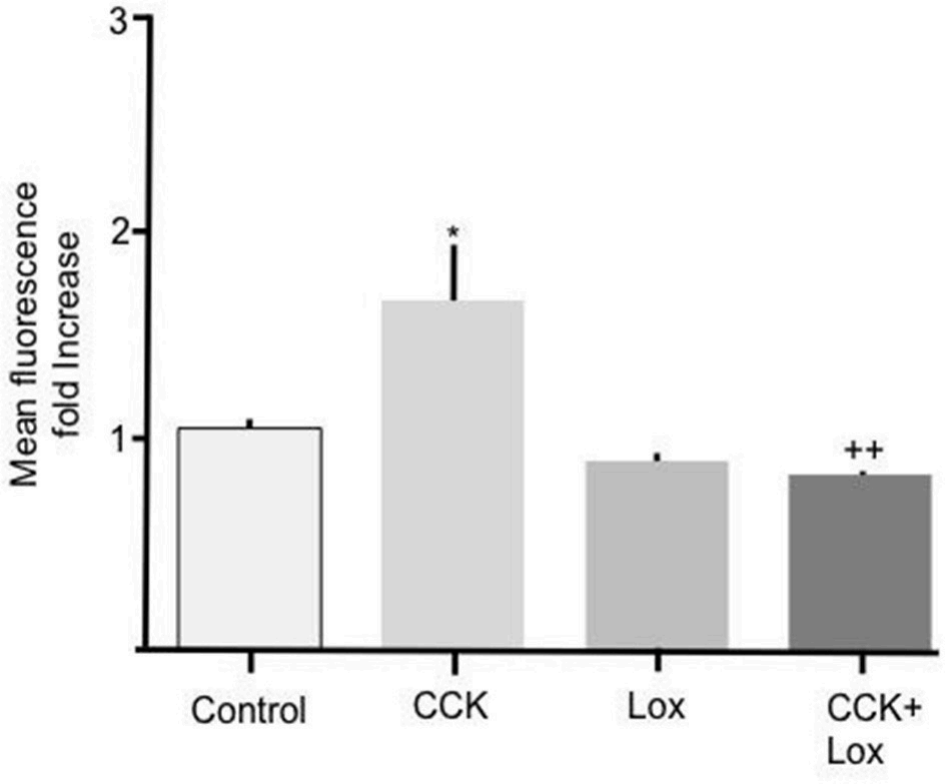
Loxiglumide inhibits the stimulatory effects of CCK on cellular Bodipy-FA uptake. The stimulatory effect of 10 pM CCK was completely inhibited by Loxiglumide (Lox) 100 μM. Incubation with loxiglumide alone caused no significant change in FA uptake when compared with control. Cells were incubated for 15 min with Lox, before application of Bodipy-FA for 2 min. Means represent replicates performed in triplicate and error bars represent ± SEM (*n* = 3) ^*^p > 0.05 compared to control; ++*p* > 0.01 compared to cells treated with CCK alone.

To determine the mechanism responsible for mediating the stimulatory effect of CCK we performed experiments to elucidate the involvement of membrane bound CCK receptors. Two isoforms of CCK receptor are responsible for mediating the action of CCK, CCK-R_A_, and CCK-R_B_. Because unsulfated CCK showed no effect on Bodipy-FA uptake we hypothesized that the CCK-R_B_ receptor, which is sensitive to non-sulfated CCK, was not involved in mediating the observed response. Therefore, the selective CCK-R_A_ antagonist, loxiglumide was employed to test the involvement of the CCK-R_A_ receptor. Alone 100 μM Loxiglumide had no significant effect on Bodipy-FA uptake, however pre-treatment of primary enterocytes with loxiglumide completely abolished the stimulatory effect conferred by CCK (*p* > 0.01, Figure 3). This implies that the CCK was acting via the CCK-R_A_.

### The mechanism by which CCK acts

**Figure 4:**
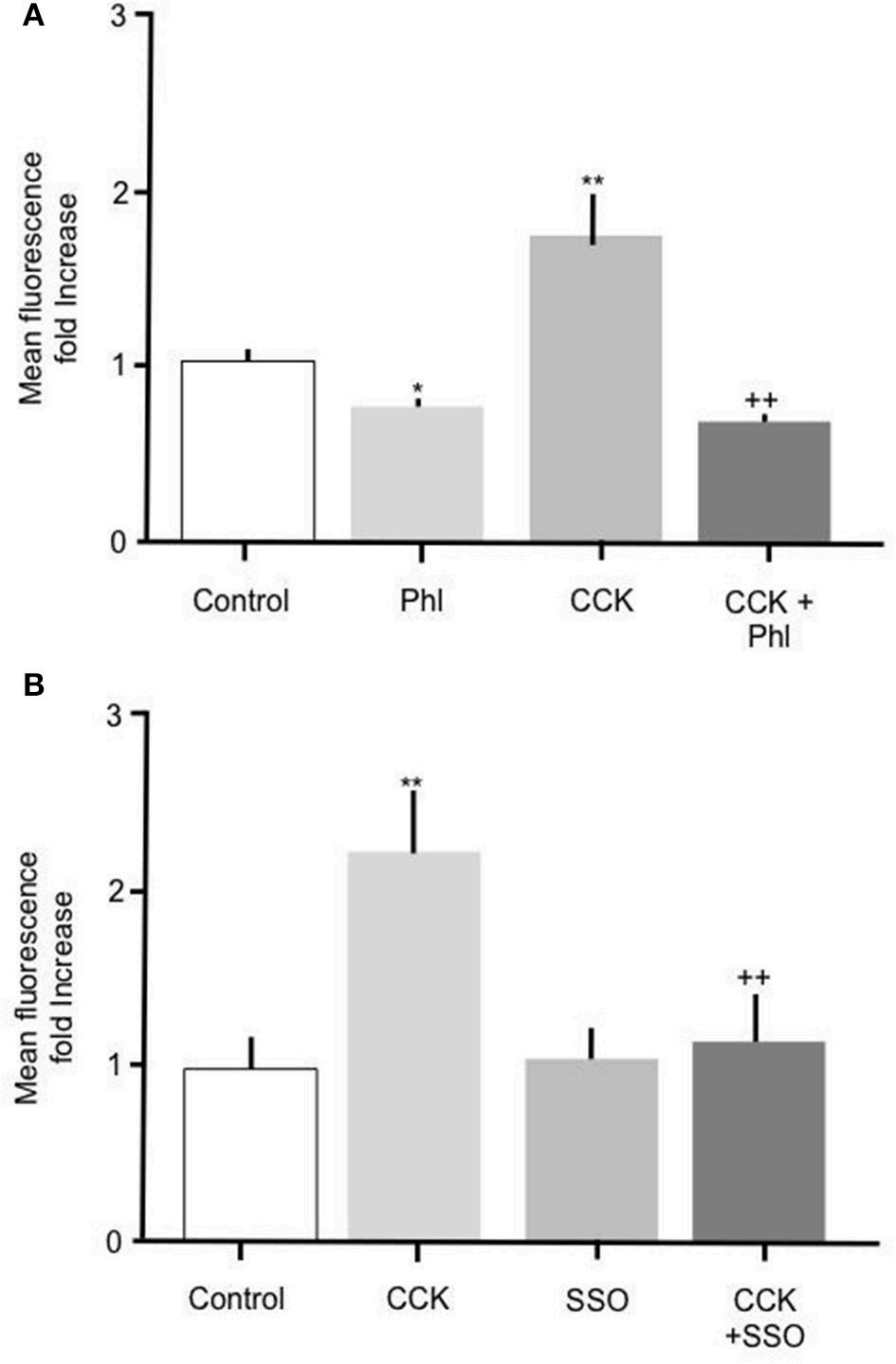
The stimulatory effect of CCK on Bodipy-FA uptake is inhibited by phloretin or SSO. **(A)** Cellular Bodipy-FA uptake was significantly increased when incubated with 10 pM CCK. This increase was abolished in the presence of 200 μM phloretin. Phloretin alone caused a significant decrease in Bodipy-FA uptake to below the control level. **(B)** The stimulatory effect of CCK was also completely inhibited in the presence of 1 μM SSO. SSO alone had no effect on mean cell fluorescence compared with control. Cells were preincubated for 15 min with either inhibitors, before application of Bodipy-FA for 2 min. Means represent replicates performed in triplicate and error bars represent SEM (*n* = 3) ^*^p > 0.05, ^**^p > 0.01 compared to control; ++*p* > 0.01 compared to CCK.

To further elucidate the mechanism mediating the CCK effect we performed experiments to determine whether carrier mediated FA transport was responsible for the observed increase in FA uptake. To do this primary enterocytes were pre-treated with known inhibitors of the FA translocase CD36. Alone, incubation of intestinal cells with 200 μM phloretin significantly reduced Bodipy-FA uptake (Figure 4A *p* > 0.01), indicating that under control conditions carrier mediated FA transport contributed to the base line FA uptake. Incubation of intestinal cells with 10 pM CCK characteristically caused a significant increase in Bodipy-FA uptake (*p* > 0.01, Figure 4A) whereas pre-treatment of cells with phloretin prior to CCK exposure negated this induced increase (*p* < 0.01). Therefore, phloretin inhibited CCK-induced Bodipy-FA uptake implicating carrier mediated FA transport in the CCK effect.

Because phloretin is a relatively broad-spectrum inhibitor of carrier-mediated transport, to confirm involvement of CD36 we pre-treated cells with 1 μM of the selective CD36 transport inhibitor SSO. Treatment with 1 μM SSO alone, unlike phloretin, did not cause Bodipy-FA uptake to decrease to below control levels (Figure 4B), but like phloretin completely abolished CCK-induced increase in Bodipy-FA uptake (Figure 4B, *p* > 0.01). Taken together, these data show that Bodipy-FA uptake induced by CCK is carrier-mediated and involves CD36.

### Upregulation of fatty acid uptake by OEA or GLP-2 is mediated via CD36

**Figure 5:**
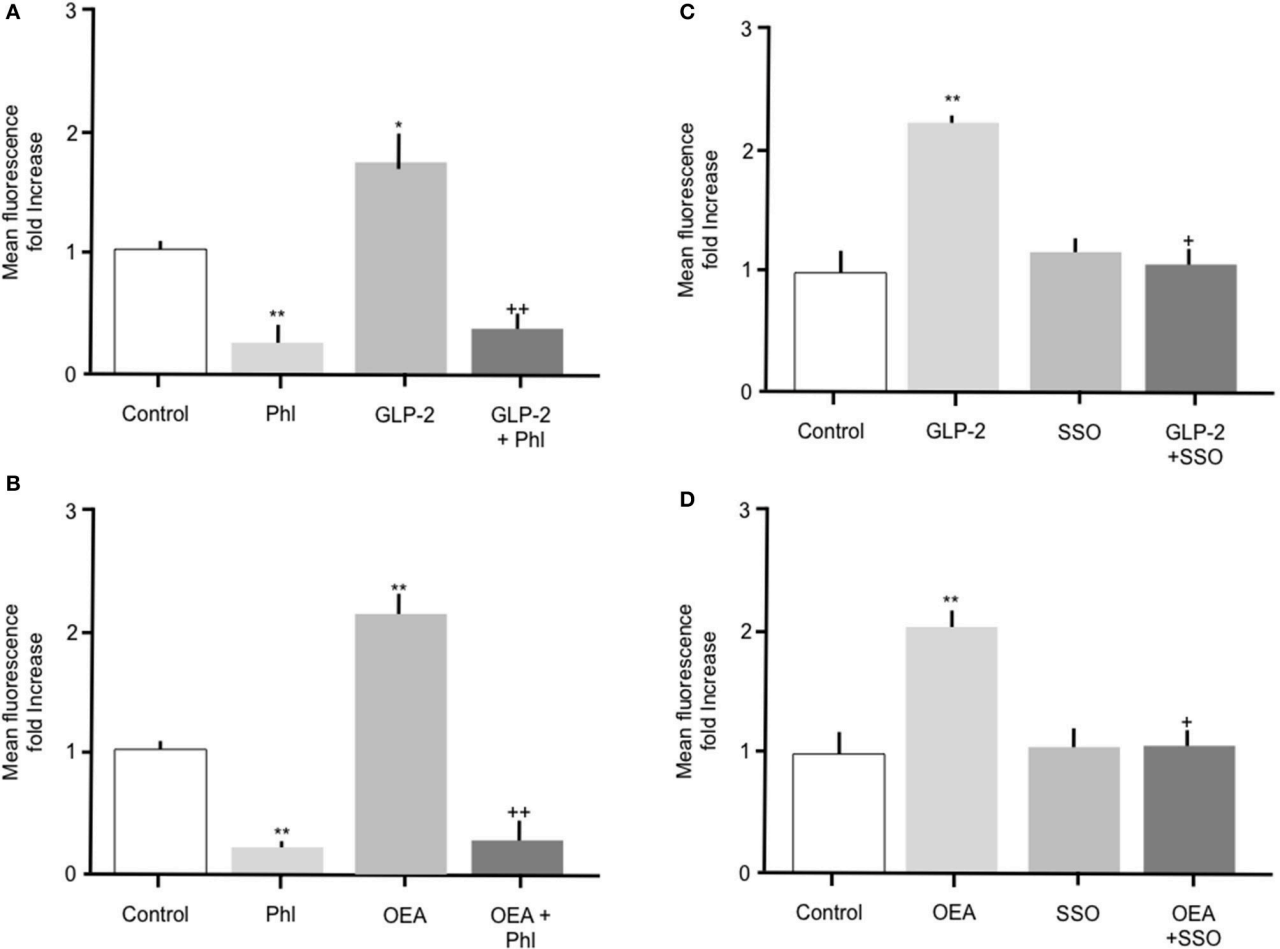
The stimulatory effects of GLP-2 or OEA on Bodipy-FA uptake into primary duodenal cells are inhibited by phloretin or SSO. **(A)** GLP-2 (10 pM) stimulated Bodipy-FA uptake into primary duodenal cells. The GLP-2 induced increase was abolished in the presence of 200 μM phloretin. Phloretin alone caused a significant decrease in Bodipy-FA uptake to below the control level. **(B)** Incubation of duodenal cells with OEA (10 nM) stimulate Bodipy-FA uptake. Phloretin completely inhibited the OEA effect and caused a significant decrease in Bodipy-FA uptake to below the control level. **(C)** GLP-2 (10 pM) induced increase was abolished in the presence of 1 μM SSO. **(D)** OEA (10 nM) stimulated Bodipy-FA uptake was completely inhibited by 1 μM SSO. Means represent replicates performed in triplicate and error bars represent SEM (*n* = 3) ^*^p > 0.05, ^**^p > 0.01 compared to control; +*p* > 0.05, ++*p* > 0.01 compared to GLP-2 or OEA.

Throughout the studies described herein, OEA or GLP-2 were included as positive controls. Incubation of intestinal cells with 10 nM OEA or 10 pM GLP-2 resulted in a significant (*p* > 0.05) increase in Bodipy-FA uptake, which was abolished by phloretin (Figure 5). As previously observed, phloretin reduced FA uptake to levels significantly below control. Incubation of intestinal cells with OEA or GLP-2 in the presence of 1 μM SSO completely inhibited the hormone-stimulated uptake of Bodipy-FA, but unlike phloretin did not reduce uptake to below control levels (Figures 5C, D). These data suggest that OEA or GLP-2 stimulated FA uptake is mediated by CD36. When primary enterocytes from CCK ko/LacZ mice were used, OEA or GLP-2 stimulated FA uptake suggesting that these agents did not require CCK to elicit their action on fatty acid uptake (data not shown).

## Discussion

The aim of the current research was to determine whether CCK modulates FA uptake by primary duodenal cells and if so to determine the mechanism of this action. Exposing mouse duodenal primary enterocytes to a physiological dose of CCK reproducibly caused a robust increase in C12 FA uptake. Irrespective of the genetic background of the mice strain, CCK reproducible induced increased FA uptake. Therefore, like GLP-2 and OEA CCK has the capacity to potentiate fatty acid uptake into enterocytes. This newly discovered function compliments the established actions of CCK that are geared for optimal and efficacious absorption of FA. Furthermore, our findings suggest that CCK released from enteroendocrine cells in the duodenum has the potential to act locally, in a paracrine manner, on neighboring enterocytes (see below).

Intriguingly, CCK showed a narrow window of activity compared to that reported by others in different tissues to duodenum (e.g., Simasko et al., 2002). We employed a CCK concentration of 10 pM because our concentration-response experiments showed this gave a statistically significant increase in Bodipy-FA uptake. Although, we observed some dose dependency, the levels of apparent increase did not reach statistical significance. At 10 pM, CCK is at the bottom end of the CCK-R_A_ binding curve although this concentration is capable of inducing near maximal increase in intracellular Ca^2+^ (Dong et al., 2014). Currently, the reasons why higher doses of CCK did not give statistically significant increases remain obscure. Taking a methodological view, conducting independent experiments for each dose would result in more concentrations being deemed statistically significant. On the other hand and from a physiological standpoint, there is evidence of CCK receptor heterogeneity (Morton et al., 2002), and CCK receptor function is modified by other cellular factors including membrane lipids (Potter et al., 2012). These reports indicate that tissue and cellular heterogeneity may underpin observed functional nuances that require careful consideration when designing future experiments to investigate CCK receptor function.

Having revealed a hitherto undescribed action of CCK we sought to determine the mechanism responsible for this effect. To do so we first determined which of the two described CCK receptors, if any, were responsible for transducing the hormone signal. These experiments showed that the selective CCK-R_A_ receptor antagonist Loxiglumide abolished the CCK-induced increase in FA uptake indicating that the CCK-R_A_ receptor was necessary for the observed response. Taken on face value this finding would suggest that the CCK-R_A_ receptor is expressed on enterocytes and CCK binding to this receptor triggered an intracellular response leading to an increase in FA uptake. The currently available expression data contained within the public databases accessed through NCBI or proprietary applications such as, Genevestigator record “medium” levels of CCK-R_A_ transcripts in mouse or human duodenum (Laule et al., 2006). Also, in humans CCK-R_A_ mRNA has been shown to be present in duodenal mucosa (Weinberg et al., 1997; Funakoshi et al., 1999). Thus, mRNA encoding CCK-R_A_ has been detected in duodenum epithelial cells, therefore corroborating our functional studies indicating the presence of the CCK-R_A_ receptor. An alternative mechanism is also plausible: It is conceivable that CCK may have acted indirectly via cells other than enterocytes and triggered release of an intermediary message that in turn propagated the observed increase in FA uptake. Considering this second possibility, the primary duodenal cells used in the current study comprised of mainly enterocytes and relatively low levels of other cell types (enteroendocrine ~1%, goblet ~10% and tuft cells ~1% Gerbe et al., 2012; Simmons et al., 2015), therefore it is possible, but untested that CCK may act via the CCK-R_A_ to stimulate release of a factor that in turn stimulated enterocytes.

Throughout the course of the experiments described herein, OEA and GLP-2 were used as positive controls to ensure viability of the primary cells in the Bodipy FA uptake assay. OEA or GLP-2 also stimulated FA uptake into enterocytes and interestingly CCK was not required for this action because when FA uptake experiments were carried out in cells from CCK knockout mice the stimulatory activity of both agents remained intact. Therefore, CCK is not a requirement for OEA or GLP-2 to bring about increase in FA uptake.

CD36 is a major potentiator of cellular FA uptake, is expressed in the intestinal epithelia (Lobo et al., 2001; Abumrad and Davidson, 2012) and is implicated in FA sensing (Nassir et al., 2007). We therefore set out to establish whether CD36 was involved in CCK-induced increased FA uptake. Two known CD36 inhibitors were employed; phloretin, a relatively broad-spectrum inhibitor and SSO, a CD36 selective inhibitor. Both reagents blocked CCK-stimulated FA uptake indicating that CD36 played a critical role in observed response (Ibrahimi et al., 1996; Coort et al., 2002; Pohl et al., 2005; Schwenk et al., 2010). Our findings fit well with those of Hsieh et al. (2009) who reported GLP-2-induced lipid uptake required CD36. Previously, CD36 has also been reported to participate in OEA-stimulated FA uptake (Yang et al., 2007) which, when considered alongside the findings of the current study and those of Hsieh et al. (2009), raises the possibility that CD36 acts as a point of integration for several gut hormones.

A consistent observation in the current study was that phloretin reduced FA uptake to below control levels whereas SSO did not. Because phloretin is relatively broad spectrum inhibitor, for example phloretin inhibits both glucose transport by SGLT proteins (Wright et al., 1991) and urea transport by UT urea transporters (Smith et al., 1995), this finding suggests that it may inhibit other FA transporters. For example, fatty acid transport protein 4 (FATP4) is expressed in duodenum and inhibited by phloretin (Masson et al., 2010; Kazantzis and Stahl, 2012) and inhibition of this transporter by phloretin likely explains the reduced uptake seen in phloretin treated samples to levels below those observed in control cells. Additionally, because SSO did not reduce uptake to below control values, CD36 is likely to be solely responsible for mediating the observed CCK-induced increase in enterocyte FA permeability.

In summary, the present work suggests that CCK plays a role in duodenal FA absorption. This conclusion raises the interesting possibility that CCK secreted by enteroendocrine cells embedded within the intestinal epithelium acts in a paracrine manner to stimulate adjacent enterocytes resulting in increased FA uptake. This is not a new concept with respect to enteroendocrine cell hormones and is one that has been suggested by others (Newberry and Davidson, 2009). Importantly, recent morphological studies have revealed structures that may facilitate this action: By utilizing exquisite imaging methods in mouse Liddle et al. have characterized granule containing processes that emanate from ileal and colonic PYY cells. These projections protrude from the basolateral surface of enteroendocrine cells and appear ideally positioned to enable paracrine communication between cells in the local environment (Bohórquez and Liddle, 2011, 2015; Bohórquez et al., 2011, 2015). Whether these structures are present in duodenum or participate in paracrine signaling remain to be determined.

The proposition that CCK induces an increase in enterocyte FA to facilitate nutrient absorption is supported by reported findings in CCK-KO mice (King et al., 2015). In response to acute intraduodenal introduction of lipids mice lacking CCK were observed to have delayed secretion of Apo B48-chylomicrons and lipid transport to the lymphatic system. Both of these effects are indicative of impaired FA uptake into enterocytes compared to wild type animals and support a physiological role for CCK-induced lipid absorption (King et al., 2015).

In conclusion, to the list of established functions of CCK can be added stimulator of duodenal enterocyte FA uptake. This action is mediated by the CCK-R_A_ receptor and CCK induced FA uptake is carrier mediated by CD36. In part our findings help explain defective lipid uptake in CCK knockout mice and are worthy of future consideration along with the reported actions of GLP-1 and OEA when considering hormone induced intestinal lipid absorption.

## Author contributions

CD: performed the majority of the experiments and processed data. Grants held by CS and JM funded the work. CS performed some experiments. JM and CS conceived the experiments. CS and JM prepared the manuscript.

### Conflict of interest statement

The authors declare that the research was conducted in the absence of any commercial or financial relationships that could be construed as a potential conflict of interest.

## Supplementary material

Supplementary Figure 1 Determination of the optimal incubation time and concentration of Bodipy-FA for uptake studies. **(A)** Optimization of Bodipy-FA concentration. Mean cell fluorescence plotted against Bodipy-FA concentration. Cells were incubated in Bodipy-FA at concentrations ranging between 2.5 and 50 μM. The EC50 was estimated to be 6.3 μM. **(B)** Incubation time optimization. Mean cell fluorescence plotted against incubation time. Cells were incubated with Bodipy-FA for periods ranging from 0.5 to 30 min. The T50 was calculated at 7.9 min. Using FACS analysis to measure fluorescence, each measurement represented 10,000 events. Means represent replicates performed in triplicate and error bars represent SEM (*n* = 3). The dash line indicates T50 and EC50 values.

Supplementary Figure 2 Representative plots showing derivation of FACS gates. Gating was set to differentiate for cell size and viability. **(A)** Dot plot of forward scatter (x-axis) vs. pulse width (y-axis). Forward scatter reflected the cell-surface size. R1 gating was employed to exclude events that did not have properties typical of cells or events of atypical cell size which represented debris or clumps of cells. **(B)** Dot plot of forward scatter (x-axis) vs. side scatter (y-axis). This plot measured cell events according to size and cell granularity. R2 gating represented the properties of single, viable cells. Red dots represent analyzed “events.”

Supplementary Figure 3 Representative histogram showing recorded FACS events for cellular Bodipy-FA uptake experiment. Within each cell sample, cellular fluorescence was measured for 10,000 events. The histogram shows Bodipy (530/40) fluorescence (x axis) vs. cell count (y-axis) for control cells (black line) and cells pre-treated with CCK (10 pM) (red line data). Treatment with CCK evoked a rightward shift in cell fluorescence representing cellular Bodipy-FA uptake.

Supplementary Figure 4 Competition of Bodipy-FA with unlabeled C12 decanoic acid inhibits cellular uptake of fluorescent Bodipy FA. Incubation of cells with Bodipy-FA with or without 250 μM unlabeled C12-decanoic acid for 2 min inhibited uptake of fluorescent Bodipy-FA. Means represent replicates performed in triplicate and error bars represent SEM (*n* = 3) ^*^*p* > 0.05, ^**^*p* > 0.01 compared to control; ++*p* > 0.01 compared to CCK.
