# Supplementary figures and images for: Sulfated Cholecystokinin-8 Promotes CD36—Mediated Fatty Acid Uptake into Primary Mouse Duodenal Enterocytes
Source: Front Physiol. 2017 Sep 1;8:660. doi: 10.3389/fphys.2017.00660 (PMC5586203; doi:10.3389/fphys.2017.00660)

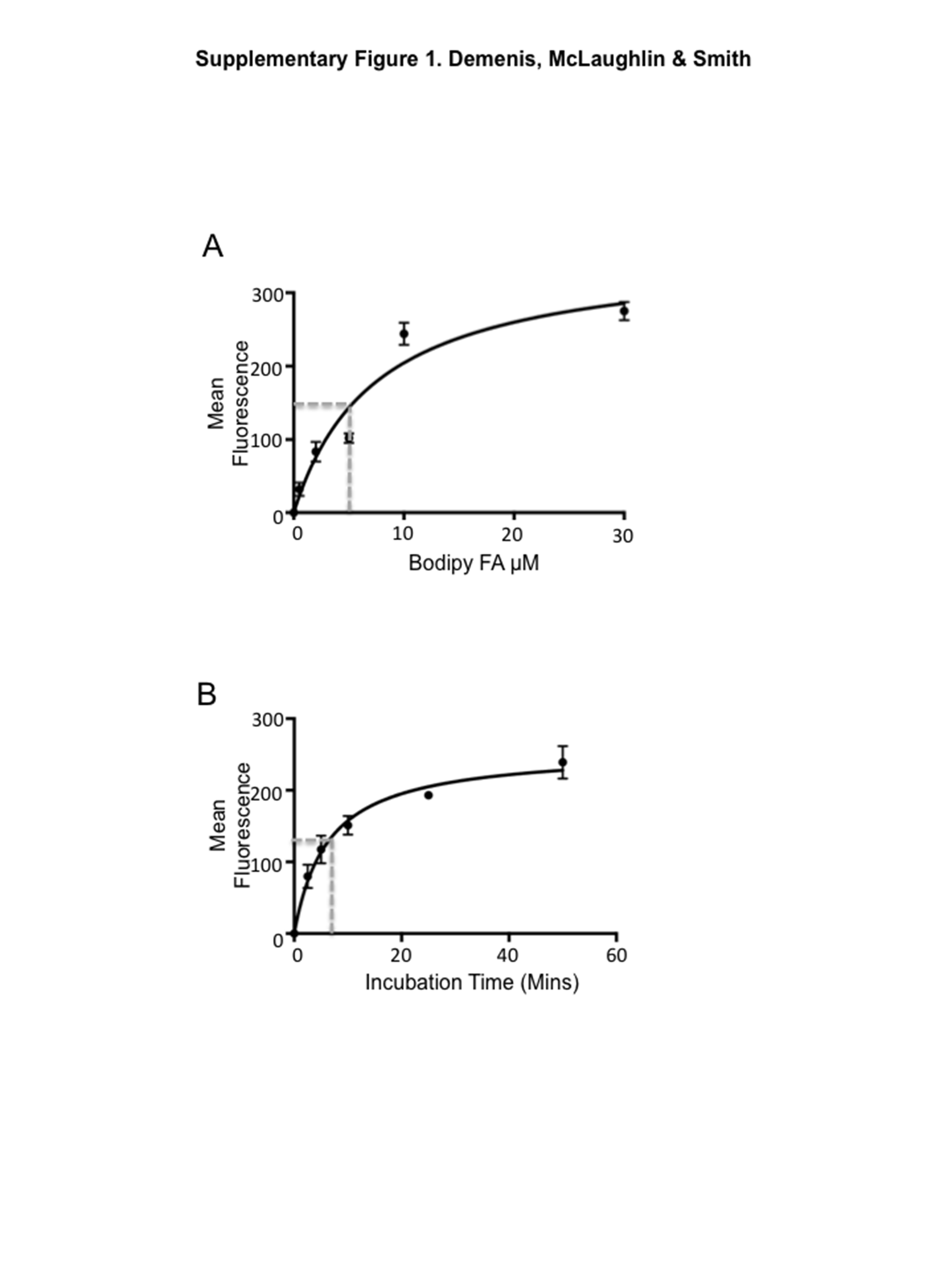

Supplement: Supplementary Figure 1 — Determination of the optimal incubation time and concentration of Bodipy-FA for uptake studies. (A) Optimization of Bodipy-FA concentration. Mean cell fluorescence plotted against Bodipy-FA concentration. Cells were incubated in Bodipy-FA at concentrations ranging between 2.5 and 50 μM. The EC50 was estimated to be 6.3 μM. (B) Incubation time optimization. Mean cell fluorescence plotted against incubation time. Cells were incubated with Bodipy-FA for periods ranging from 0.5 to 30 min. The T50 was calculated at 7.9 min. Using FACS analysis to measure fluorescence, each measurement represented 10,000 events. Means represent replicates performed in triplicate and error bars represent SEM (n = 3). The dash line indicates T50 and EC50 values. [file Image1.tiff]

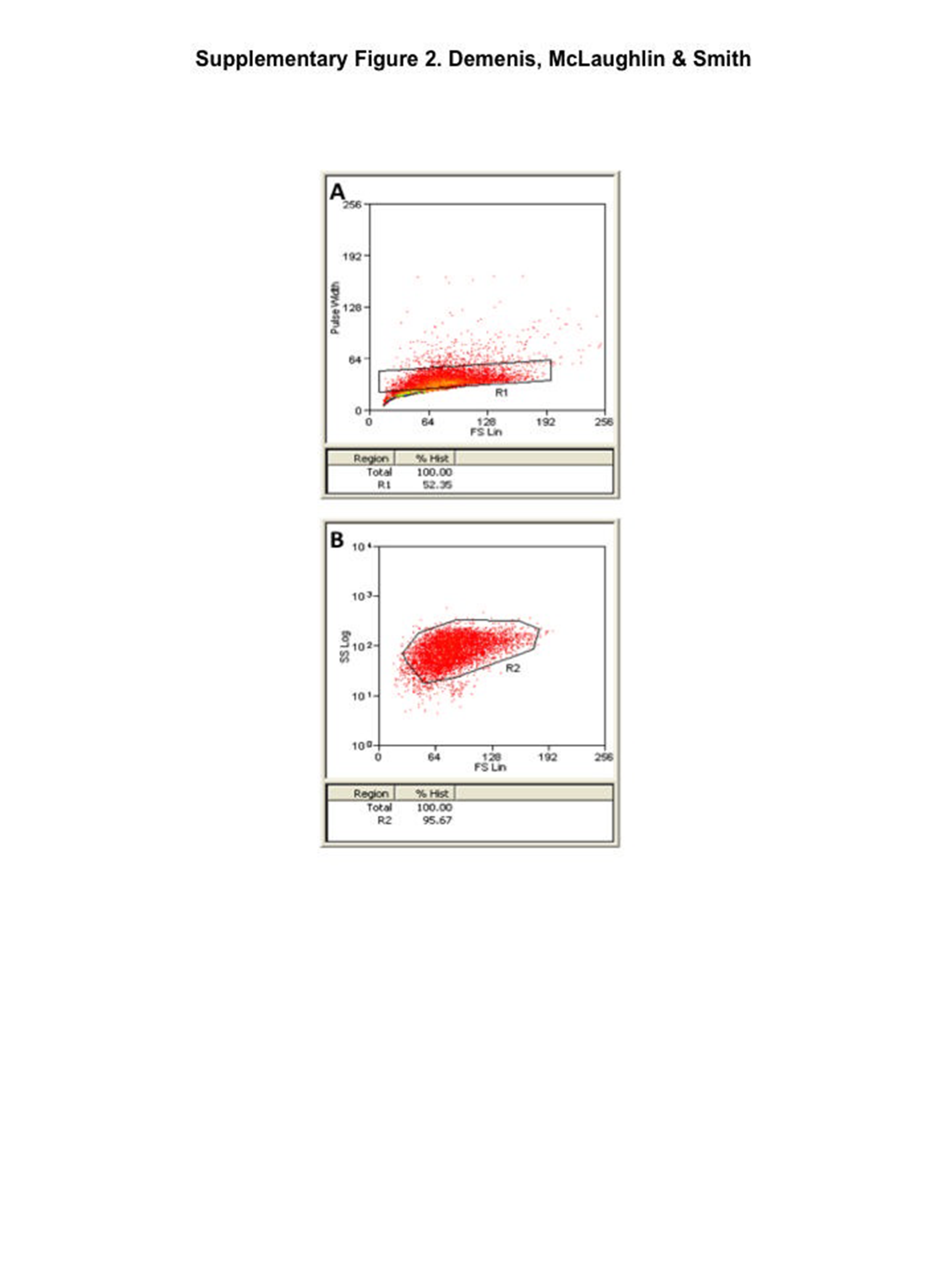

Supplement: Supplementary Figure 2 — Representative plots showing derivation of FACS gates. Gating was set to differentiate for cell size and viability. (A) Dot plot of forward scatter (x-axis) vs. pulse width (y-axis). Forward scatter reflected the cell-surface size. R1 gating was employed to exclude events that did not have properties typical of cells or events of atypical cell size which represented debris or clumps of cells. (B) Dot plot of forward scatter (x-axis) vs. side scatter (y-axis). This plot measured cell events according to size and cell granularity. R2 gating represented the properties of single, viable cells. Red dots represent analyzed “events.” [file Image2.tiff]

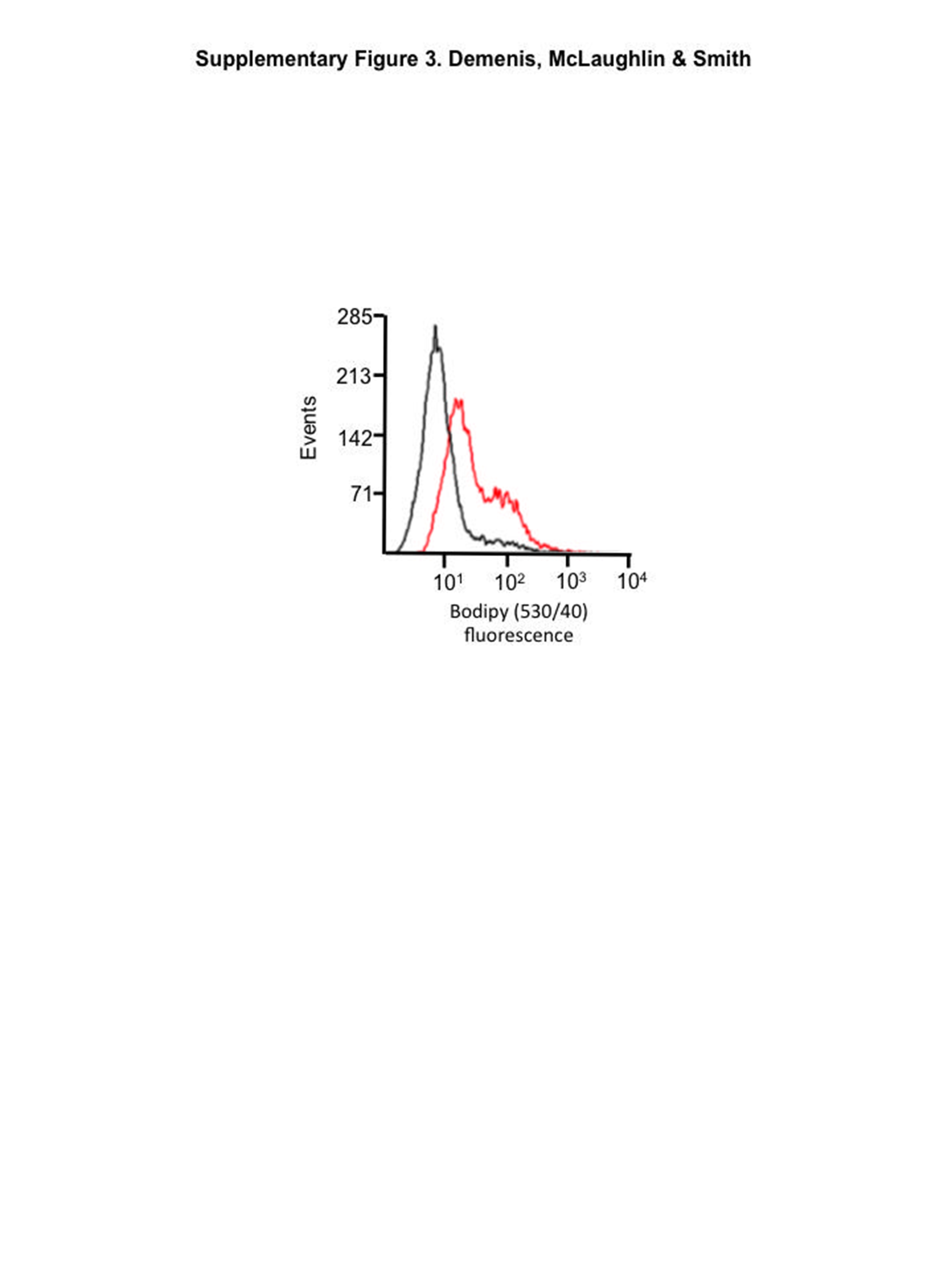

Supplement: Supplementary Figure 3 — Representative histogram showing recorded FACS events for cellular Bodipy-FA uptake experiment. Within each cell sample, cellular fluorescence was measured for 10,000 events. The histogram shows Bodipy (530/40) fluorescence (x axis) vs. cell count (y-axis) for control cells (black line) and cells pre-treated with CCK (10 pM) (red line data). Treatment with CCK evoked a rightward shift in cell fluorescence representing cellular Bodipy-FA uptake. [file Image3.tiff]

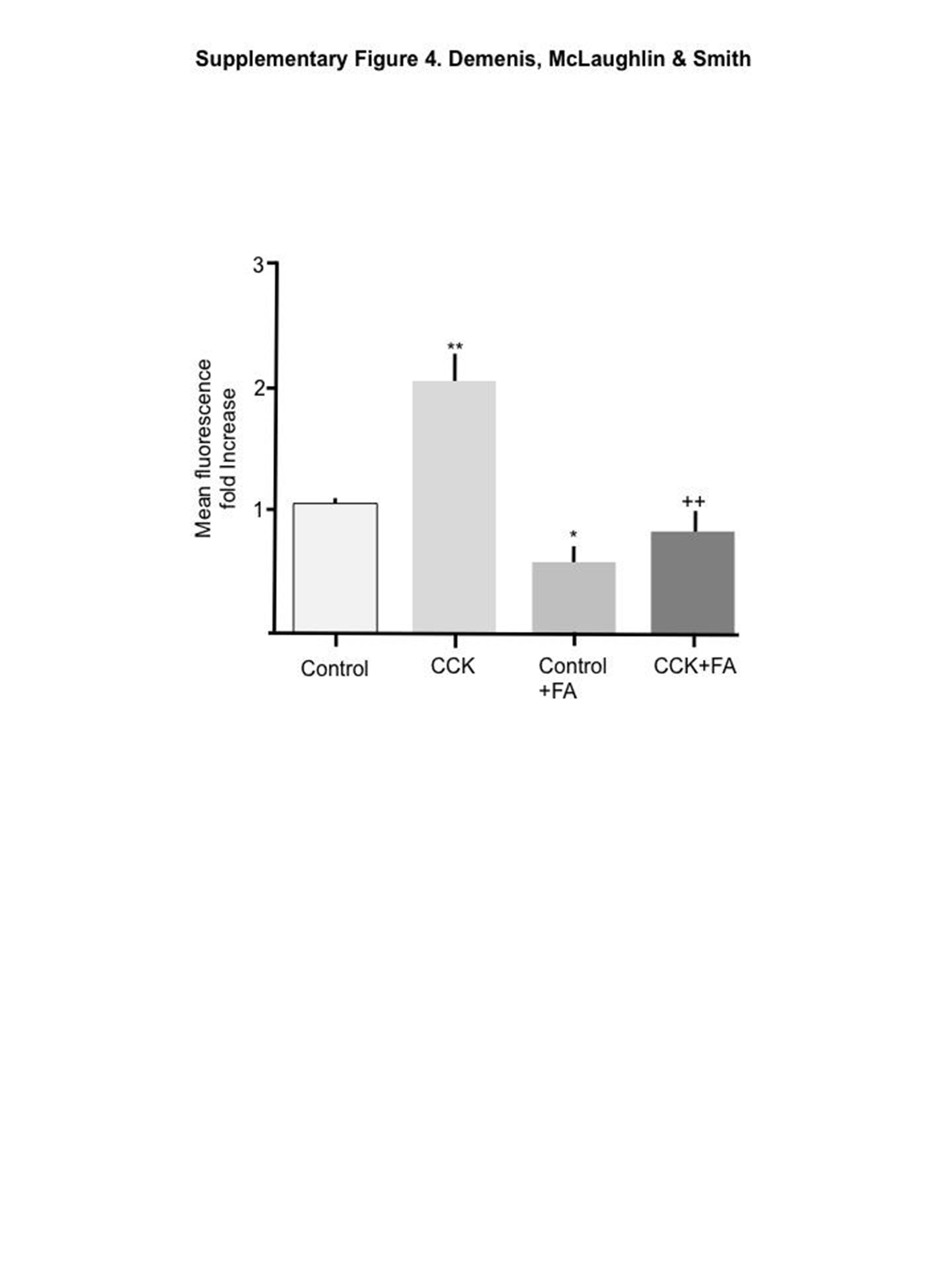

Supplement: Supplementary Figure 4 — Competition of Bodipy-FA with unlabeled C12 decanoic acid inhibits cellular uptake of fluorescent Bodipy FA. Incubation of cells with Bodipy-FA with or without 250 μM unlabeled C12-decanoic acid for 2 min inhibited uptake of fluorescent Bodipy-FA. Means represent replicates performed in triplicate and error bars represent SEM (n = 3) *p > 0.05, **p > 0.01 compared to control; ++p > 0.01 compared to CCK. [file Image4.tiff]
